# Influence of head‐up tile and lower body negative pressure on the internal jugular vein

**DOI:** 10.14814/phy2.15248

**Published:** 2022-05-17

**Authors:** Shigehiko Ogoh, Ai Hirasawa, Shigeki Shibata

**Affiliations:** ^1^ Department of Biomedical Engineering Toyo University Saitama Japan; ^2^ Neurovascular Research Laboratory Faculty of Life Sciences and Education University of South Wales Pontypridd UK; ^3^ Department of Health and Welfare Faculty of Health Sciences Kyorin University Tokyo Japan; ^4^ Department of Physical Therapy Faculty of Health Science Kyorin University Tokyo Japan

**Keywords:** cerebral venous outflow, collapse, head‐up tilt, lower body negative pressure, orthostatic stress

## Abstract

Head‐up tilt (HUT)‐induced gravitational stress causes collapse of the internal jugular vein (IJV) by decreasing central blood volume and through mass‐effect from the surrounding tissues. Besides HUT, lower body negative pressure (LBNP) is used to stimulate orthostatic stress as an experimental model. Compared to HUT, LBNP has less of a gravitational effect because of the supine position; therefore, we hypothesized that LBNP causes less of a decrease in the cross‐sectional area of the IJV compared to HUT. We tested the hypothesis by measuring the cross‐sectional area of the IJV using B‐mode ultrasonography while inducing orthostatic stress at levels of −40 mmHg LBNP and 60° HUT. The cross‐sectional area of IJV decreased from the resting baseline during both LBNP and HUT trials, but the LBNP‐induced decrease in the cross‐sectional area of IJV was smaller than that of HUT (right, −45% ± 49% vs. −78% ± 27%, *p* = 0.008; left, −49% ± 27% vs. −78% ± 20%, *p* = 0.004). Since changes in venous outflow may affect cerebral arterial circulation, the findings of the present study suggest that orthostatic stress induced by different techniques modulates cerebral blood flow regulation through its effect on venous outflow.

## Graphical abstract

Orthostatic tolerance has been investigated with large fluid shifts using lower body negative pressure (LBNP) and head‐up tilt (HUT). HUT‐induced gravitational stress also leads to the collapse of the internal jugular vein (IJV) by decreasing central blood volume and through mass‐effect from the surrounding tissues. However, changes in cross‐sectional area of IJV were less profound when orthostatic stress was induced by LBNP compared to HUT. This phenomenon may need to be considered when evaluating cerebral blood flow regulation using LBNP or HUT since IJV blood flow affects arterial cerebral blood flow regulation.

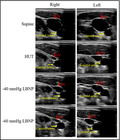

## 1 INTRODUCTION

Tolerance to orthostatic stress requires adequate cardiovascular control, including an adequate neurohumoral reflex response to central blood volume, to avoid syncope (Cooper & Hainsworth, 2001, 2002; Ogoh et al., 2003). Microgravity from Earth or Earth from Microgravity induces drastic fluid shifts that modify the cardiovascular response of astronauts and consequently impair orthostatic tolerance (Bondar et al., 1994; Fu et al., 2002, 2019). Therefore, the mechanism of determining orthostatic tolerance is important, especially in astronauts (Levine, 1993). Orthostatic tolerance has been investigated through ground‐based studies by inducing large fluid shifts using lower body negative pressure (LBNP) and head‐up tilt (HUT). Some studies (Han et al., 2009; Kaur et al., 1985; Kuriyama et al., 2000; Ogoh et al., 2013, 2015) investigated cerebral blood flow (CBF) regulation as a surrogate of orthostatic tolerance using these experimental techniques. Generally, it has been thought that the orthostatic stress during HUT trial is matched with that of LBNP trial, as an experimental model, that induces the same change in fluid shifts (Bronzwaer et al., 1985).

Yet, on the contrary, it is noteworthy that HUT‐induced gravitational stress also leads to the collapse of the internal jugular vein (IJV) by decreasing central blood volume and through mass‐effect from the surrounding tissues (Dawson et al., 2004). Although it is unclear how this phenomenon contributes to CBF regulation, the venous outflow is associated with CBF regulation (Ogoh et al., 2016, 2020; Sato et al., 2017). Thus, it is possible that a gravitationally stress‐induced change in venous outflow may affect CBF regulation. LBNP causes fluid shifts similarly to HUT, but it has less of a gravitationally‐induced effect on the IJV because it maintains a supine position. Therefore, we hypothesized that LBNP causes a smaller decrease in the cross‐sectional area of the IJV as compared to HUT. This investigation is important because the various morphologic changes of the IJV in response to orthostatic stress may impact CBF differently, even with the same amount of orthostatic stress. To test this hypothesis we measured the changes in the cross‐sectional area of the IJV during orthostatic stress‐induced after levels of −40 mmHg LBNP and 60° HUT.

## 2 METHODS

### 2.1 Participants

Eight healthy participants (five men and three women, age 22.6 ± 1.1 years, height 166.9 ± 6.0 cm, weight 60.8 ± 13.8 kg; mean ± standard deviation [SD]) participated in the study. The subjects underwent a medical examination, including a detailed history, and did not have any cardiovascular, pulmonary or kidney disease. The subjects were asked to abstain from caffeinated beverages, strenuous physical activity, and alcohol for 24 h before the experiment. The protocol was approved by the Ethical Committee for Human Research at Kyorin University (no. 2020‐3), and each subject provided written informed consent to participate according to the principles of the Declaration of Helsinki.

### 2.2 Experimental protocol

Participants rested in the supine position for at least 20 min before the HUT trial on the hospital tilt bed, after which they were placed at 60° HUT. Doppler measurement was performed at two minutes. Following the HUT trial, participants were positioned in the LBNP box, which was sealed at the level of the iliac crest. Participants were rested in the supine position once again while in the LBNP box for at least 20 min before initiating the LBNP trial. The subjects were asked to rest on the hospital tilt bed to allow for hemodynamics to return to baseline. Subsequently, the participants underwent −40 and −60 mmHg LBNP trials at random. Doppler measurement was performed at 2 min. Step change was performed between the two LBNP trials without rest.

### 2.3 Doppler measurement

**FIGURE 1:**
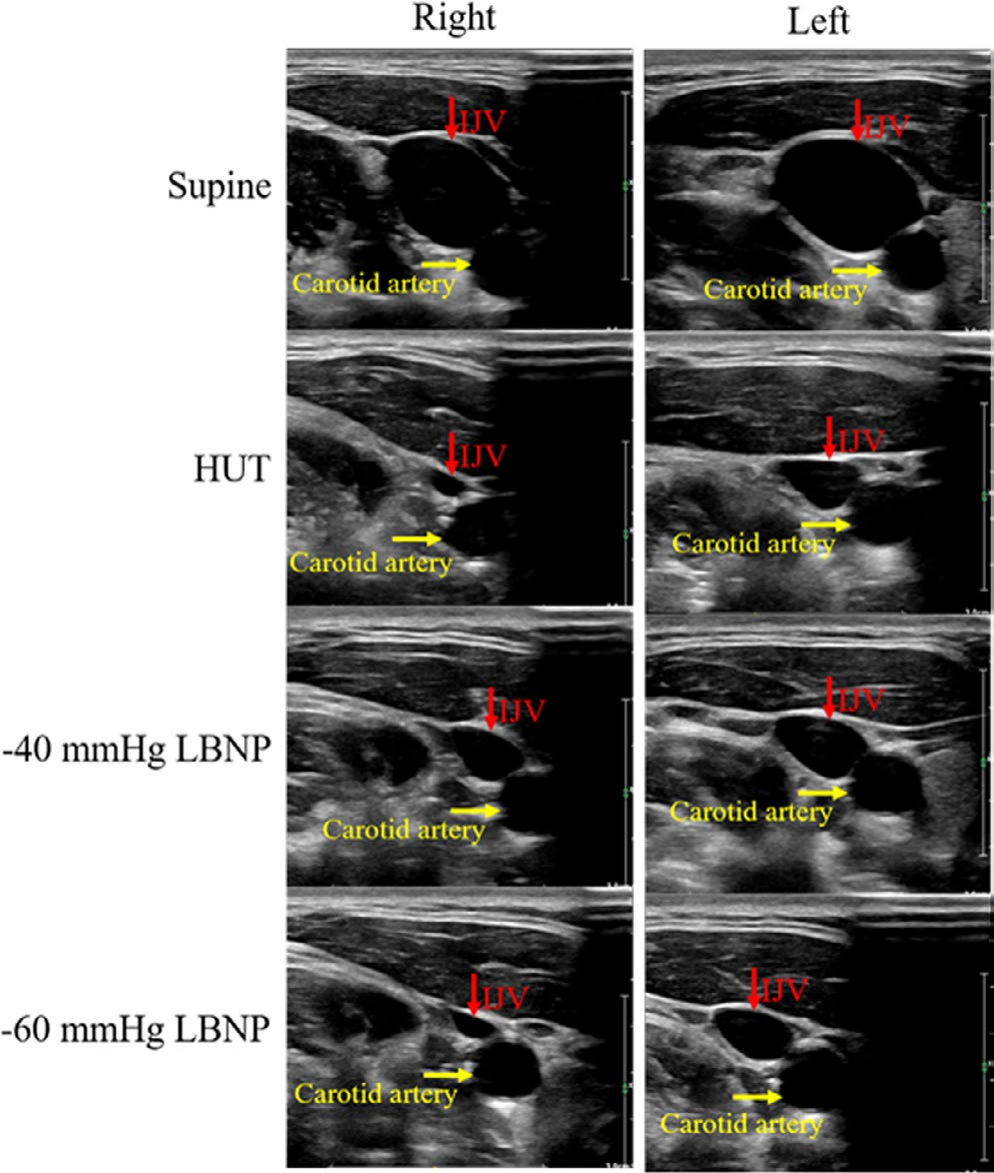
Internal jugular vein (IJV) imaging by B‐mode ultrasonography. Right and left side IJV in the supine position (top panel), 60° head‐up tilt (HUT, second top panel), −40 mmHg lower body negative pressure (LBNP, second bottom panel), and −60 mmHg LBNP (bottom panel)

The cross‐sectional area of the IJV was measured bilaterally by investigators trained in the use of B‐mode ultrasound systems (EPIQ 7; Philips) equipped with 12‐MHz linear transducers (Figure 1). The cross‐sectional area of IJV was assessed at the J3 level (as cranially as possible in the IJV after its passage through the jugular foramen). Subjects maintained their heads in the neutral position as much as possible during ultrasonography to minimize distortion of the vein by cervical rotation (Dawson et al., 2004). It was ensured that the probe position and insonation angle were consistent, and veins were not collapsed by the ultrasonographer. The cross‐sectional area of the IJV was calculated by manually measuring the image offline. Because of the fluctuation of the cross‐sectional area of the IJV caused by respiration, the largest cross‐sectional area of the IJV was measured under spontaneous respiration.

### 2.4 Cardiopulmonary measurements

Heart rate was monitored using three‐lead electrocardiography (BSM2301; Nihon Kohden). Continuous arterial blood pressure was monitored using finger photoplethysmography (Finometre MIDI; Finapres Medical System), and used to calculate mean arterial pressure (MAP). Stroke volume (SV) and cardiac output were determined from the ABP waveform using the Modelflow software program, which incorporates the sex, age, height, and weight of the subject (Beat Scope1.1; Finapres Medical Systems). The partial pressure of carbon dioxide (PetCO_2_) was measured using a capnometer (CO_2_ Monitor OLG‐2800A; Nihon Kohden).

### 2.5 Statistical analysis

Values are expressed as mean ± SD. Differences in variables between trials were compared by one‐way repeated‐measures analysis of variance with Student–Newman–Keuls post hoc test (SPSS, version 19.0; IBM). The level of significance was set at a *p*‐value of <0.05.

## 3 RESULTS

Both methods of inducing orthostatic stress caused tachycardia, increased MAP, and decreased SV and PetCO_2_ (Table 1). These changes were enhanced from −40 mmHg LBNP or HUT to −60 mmHg LBNP. Importantly, there were no significant differences in SV between −40 mmHg LBNP and 60° HUT (−19.1% ± 15.1% and −23.2% ± 10.5% from baseline, *p* = 0.156). The −60 mmHg LBNP further decreased SV from −40 mmHg LBNP and HUT (*p* < 0.001, −35.7% ± 12.3% from baseline).

**TABLE 1:** Haemodynamic response to head‐up tilt (HUT) and lower body negative pressure (LBNP)

	**Supine**	**HUT**	**LBNP**	
			**−40 mmHg**	**−60 mmHg**
HR (bpm)	77.8 ± 13.1	93.7 ± 12.0*	81.9 ± 13.8^#^	94.7 ± 17.0* ^,^ ^$^
MAP (mmHg)	73.3 ± 9.0	80.7 ± 7.5*	83.6 ± 14.1*	83.4 ± 11.4*
SV (mL)	67.2 ± 17.5	54.0 ± 17.6*	52.1 ± 16.9*	43.5 ± 16.1* ^,^ ^#^ ^,^ ^$^
PetCO^2^ (mmHg)	38.5 ± 1.6	36.7 ± 2.9*	38.1 ± 2.0^#^	36.2 ± 2.9* ^,^ ^$^

Note

Mean ± SD.

Abbreviations: HR, heart rate; MAP, mean arterial pressure; PetCO_2_, partial pressure of carbon dioxide; SV, stroke volume.

* *p* < 0.05 versus supine.

# *p* < 0.05 versus HUT.

$ *p* < 0.05 versus −40 mmHg LBNP.

**FIGURE 2:**
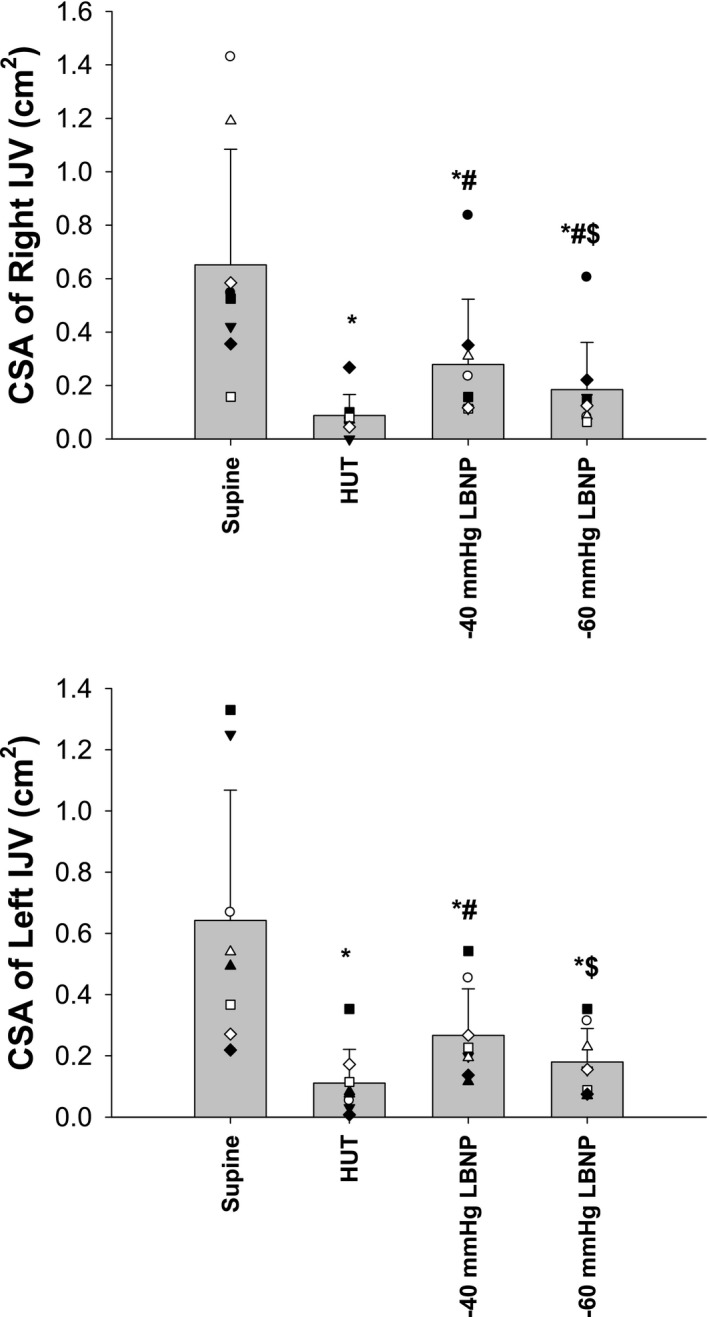
Cross‐sectional area (CSA) of the right internal jugular vein (IJV, top panel) and left IJV (bottom panel) at the supine position, 60° head‐up tilt (HUT), −40 mmHg lower body negative pressure (LBNP), and −60 mmHg LBNP. *p < 0.05 vs. supine, ^#^ *p* < 0.05 vs. HUT, ^$^ *p* < 0.05 vs. −40 mmHg LBNP

Both methods of inducing orthostatic stress decreased the cross‐sectional area of the IJV, but −40 mmHg LBNP induced a smaller bilateral decrease in the cross‐sectional area of IJV than that induced by HUT (right, −45% ± 49% vs. −78% ± 27%, *p* = 0.008; left, −49% ± 27% vs. −78% ± 20%, *p* = 0.004; Figure 2). Moreover, −60 mmHg LBNP enhanced the decrease in the cross‐sectional area of the IJV compared to −40 mmHg, but the cross‐sectional area during −60 mmHg was still larger than that of HUT.

## 4 DISCUSSION

The present study assessed the cross‐sectional area of the IJV during the LBNP and HUT trials. The novel finding is a smaller decrease in the cross‐sectional area of the IJV during the −40 mmHg LBNP compared to the 60° HUT despite a similar decrease in SV (similar orthostatic stress). Gravitational effects during orthostatic stress induce IJV collapse and decrease the cross‐sectional area of the IJV. Importantly, venous outflow affects cerebral arterial circulation; therefore, the findings of the present study suggest that the different methods of inducing orthostatic stress may modify CBF regulation via different mechanical influences on venous shape (venous outflow) despite the same systemic hemodynamics. Therefore, this phenomenon may need to be taken into consideration when studying CBF regulation using LBNP or HUT.

Both LBNP and HUT reduce venous return, and thus central blood volume, by pooling blood in the lower parts of the body (Cai et al., 1985). The LBNP at −40 mmHg produces a shift of 500–600 ml of blood into the lower extremities (Musgrave et al., 1969), which is quantitatively similar to the volume shift observed by the upright posture or 60° HUT (Perko et al., 1993; Sjostrand, 1953). Both LBNP and HUT produce a similar decline in thoracic volume and increase in pelvic and lower extremity volume as measured using impedance plethysmography (Taneja et al., 2007). In the present study, the −40 mmHg LBNP and 60° HUT led to similar changes in SV; thus, cardiac output without arterial hypotension is consistent with data from previous studies (Kitano et al., 1985; Wolthuis et al., 1974). Therefore, these findings support the contention that 60° HUT and −40 mmHg LBNP evoke a similar degree of central hypovolemia with comparable systemic hemodynamic responses.

In giraffes, since the head is 2 m above the heart, arterial blood pressure at heart level should be 155 mmHg higher than that at the head by estimating via hydrostatic pressure, however, actual arterial blood pressure at heart level was much lower (Mitchell et al., 2006). Thus, previous studies (Badeer, 1988; Hicks & Badeer, 1989) suggested that the giraffe cranial circulation can be regarded as an inverted U‐tube that functions as a siphon for supporting arterial blood flow to the brain. The pressure gradient down the jugular vein should be negative if the jugular vein acted as a siphon, however, it was the opposite in giraffes: pressure at the top of their jugular vein was far higher than that at the bottom (Hargens et al., 1987). These findings suggest at least that the principle of the siphon is not valid in giraffes. In addition, previous human studies (Dawson et al., 2004; Gisolf et al., 2005) concluded that a siphon does not operate in the cranial circulation of standing humans because orthostatic stress decreases the cross‐sectional area of the IJV and leads to IJV collapse. The IJV pressure is close to zero, maybe with, occasionally, small negative pressures probably reflecting that there are established “pockets” of blood in a vein that is collapsed at other places. In the present study, similarly, both LBNP and HUT decreased the cross‐sectional area of the IJV. This reduction is due to the gravitational influence on the tissue around the IJV (Dawson et al., 2004). This phenomenon is likely to impede anterior CBF drainage, but it has been reported that vertebral vein (VV) blood flow increases accordingly as the IJV blood flow decreases during orthostatic stress (Valdueza et al., 2000). This important finding suggests that VV blood flow is important for maintaining anterior CBF drainage and likely compensates for the decrease in IJV blood flow during orthostatic stress. The VV only compensates only for up to 50% of the reduction in IJV blood flow because of its smaller caliber, but other veins, such as the epidural veins, also drain the brain and help to regulate CBF during orthostatic stress. The orthostatic stress‐induced alteration in venous drainage distribution affects arterial CBF regulation, especially in the posterior cerebral circulation (Ogoh et al., 2016, 2020; Sato et al., 2017).

In the present study, the cross‐sectional area of the IJV responded differently to the method used to induce orthostatic. These differences in the present study are relatively small; however, it should be considered that the different methods themselves, rather than orthostatic stress itself, may modify CBF regulation. Moreover, altered venous hemodynamics may play a role in the late stages of post‐ischemic cerebral edema in patients with midline dislocation (Stolz et al., 2002), and cranial venous outflow abnormalities may cause cerebral edema after ischemic stroke (Van Lieshout et al., 1985). These findings suggest that venous drainage is important for maintaining cerebral homeostasis in patients with cerebral disease and highlight the interplay between cerebral venous outflow and CBF regulation.

### 4.1 Limitations

This study has several limitations. The IJV pressure may need to be measured to accurately identify venous collapse because the decrease in the cross‐sectional area may not be consistent through the entire length of the IJV (Dawson et al., 2004). In the present study, we measured the cross‐sectional area at only one site of IJV (at the J3 level as cranially as possible in the IJV after its passage through the jugular foramen). Therefore, our data may over‐ or under‐estimate the difference in response of the cross‐sectional area or collapse of the IJV to LBNP and HUT. If the collapse of the IJV occurs, the cross‐sectional area at one site of the IJV may not be zero, but the blood pressure is zero. Finally, the change in the cross‐sectional area of IJV should be easy to fix using M‐mode. However, it is difficult for determining “collapse” for M‐mode because the evaluation is on a straight line on the M‐mode. In addition, the respiratory fluctuation can be confirmed even on B‐mode. Thus, in the present study, the cross‐sectional area of the IJV was measured using B‐mode ultrasound systems.

## 5 CONCLUSIONS

LBNP, which stimulates orthostatic stress as an experimental model, caused different alterations in anterior venous outflow that may affect CBF regulation compared with HUT. Thus, in studying CBF regulation, it should be considered that the various methods of inducing orthostatic stress may modify CBF response independently of the orthostatic stress that is induced.

## CONFLICT OF INTEREST

No conflicts of interest, financial or otherwise, are declared by the authors.

## AUTHOR CONTRIBUTIONS

Shigehiko Ogoh and Shigeki Shibata conception and design of research; Shigehiko Ogoh, Ai Hirasawa, and Shigeki Shibata performed experiments; Ai Hirasawa analyzed data; Shigehiko Ogoh and Ai Hirasawa interpreted results of experiments; Shigehiko Ogoh prepared figures; Shigehiko Ogoh drafted the manuscript; all authors edited and revised manuscript; all authors approved the final version of the manuscript.

## Data Availability

The data that support the findings of this study are available from the corresponding author, [SO], upon reasonable request.
