# Corrigendum: Influence of head‐up tilt and lower body negative pressure on the internal jugular vein

**DOI:** 10.14814/phy2.15438

**Published:** 2022-09-06

The authors of the article by Shigehiko Ogoh et al. (2022) noticed an error in the title of their article. The word “tile” should be “tilt.” The correct article title should be Influence of head‐up tilt and lower body negative pressure on the internal jugular vein.

## References

[phy215438-bib-0001] Ogoh, S. , Hirasawa, A. , & Shibata, S. (2022). Influence of head‐up tilt and lower body negative pressure on the internal jugular vein. Physiological Reports, 10, e15248. 10.14814/phy2.15248 35581747 PMC9114655

